# Supplementary material for: Di- and Triselenoesters—Promising Drug Candidates for the Future Therapy of Triple-Negative Breast Cancer
Source: Int J Mol Sci. 2024 Jul 16;25(14):7764. doi: 10.3390/ijms25147764 (PMC11277004; doi:10.3390/ijms25147764)
Supplement: Supplementary file 1 [file ijms-25-07764-s001.zip › ijms-2975084-supplementary.pdf]

Supplementary Materials for

Di- and Triselenoesters - Promising Drug Candidates For The Future Therapy of Triple-Negative Breast Cancer

submitted by Dominika Radomska [1,*], Robert Czarnomysy [1,*], Anna Szymanowska [2], Dominik Radomski [1], Magda Chalecka [3], Arkadiusz Surazynski [3], Enrique Domínguez-Álvarez [4], Anna Bielawska [5] and Krzysztof Bielawski [1]

[1] Department of Synthesis and Technology of Drugs, Medical University of Bialystok, Kilinskiego 1, 15-089 Bialystok, Poland
[2] Department of Experimental Therapeutics, The University of Texas MD Anderson Cancer Center, Houston, TX 77054, USA
[3] Department of Medicinal Chemistry, Medical University of Bialystok, Mickiewicza 2D, 15-222 Bialystok, Poland
[4] Instituto de Química Orgánica General (IQOG-CSIC), Consejo Superior de Investigaciones Científicas, Juan de la Cierva 3, 28006 Madrid, Spain
[5] Department of Biotechnology, Medical University of Bialystok, Kilinskiego 1, 15-089 Bialystok, Poland

* Correspondence: dominika.radomska@umb.edu.pl; Tel: 0048 85 748 57 00; Fax: 0048 85 879 57 00 (D.R.); robert.czarnomysy@umb.edu.pl (R.C.)

The following document contains representative native membrane pictures from the western blot that were utilized in creating Figures 13 and 18.

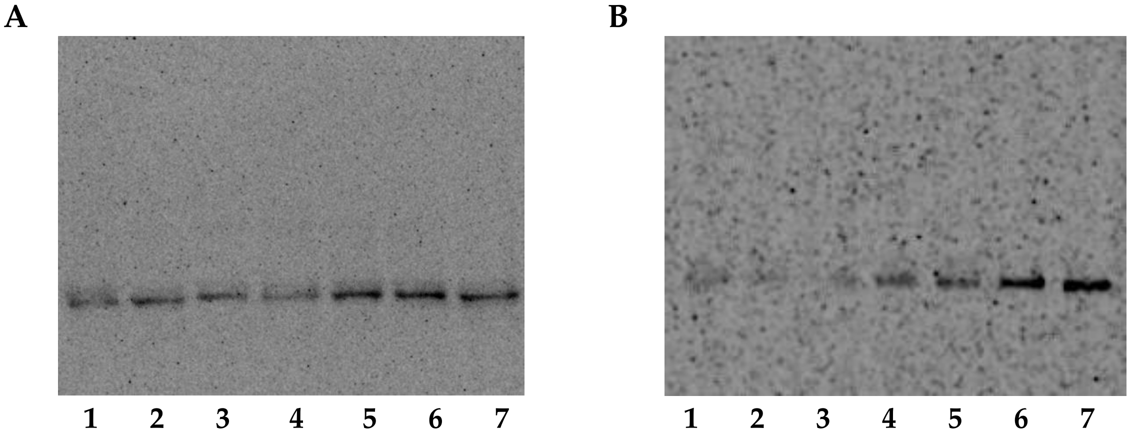

Figure S1. Original western blot membranes used for the evaluation of Bax expression in MCF-7 (**A**) and MDA-MB-231 (**B**) breast cancer cells. 1) Control; 2) Cisplatin 0.5 μM; 3) Cisplatin 1 μM; 4) EDAG-1 0.5 μM; 5) EDAG-1 1 μM; 6) EDAG-8 0.5 μM; 7) EDAG-8 1 μM.

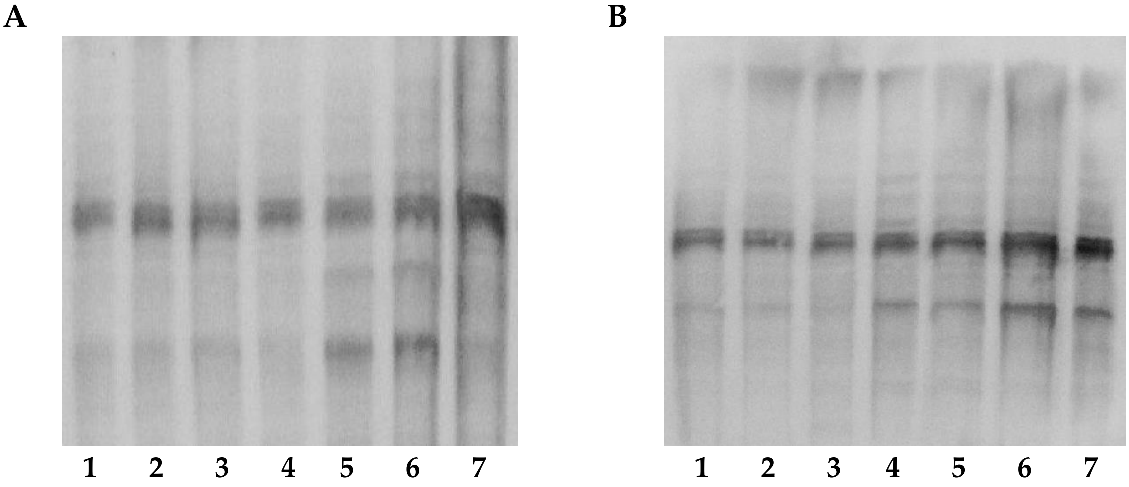

Figure S2. Original western blot membranes used for the evaluation of caspase 7 expression in MCF-7 (**A**) and MDA-MB-231 (**B**) breast cancer cells. 1) Control; 2) Cisplatin 0.5 μM; 3) Cisplatin 1 μM; 4) EDAG-1 0.5 μM; 5) EDAG-1 1 μM; 6) EDAG-8 0.5 μM; 7) EDAG-8 1 μM.

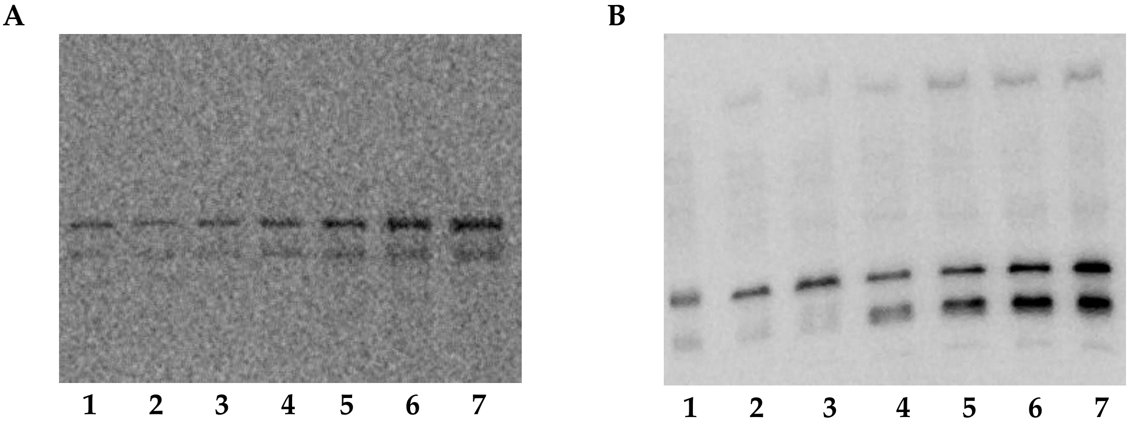

Figure S3. Original western blot membranes used for the evaluation of caspase 9 expression in MCF-7 (**A**) and MDA-MB-231 (**B**) breast cancer cells. 1) Control; 2) Cisplatin 0.5 μM; 3) Cisplatin 1 μM; 4) EDAG-1 0.5 μM; 5) EDAG-1 1 μM; 6) EDAG-8 0.5 μM; 7) EDAG-8 1 μM.

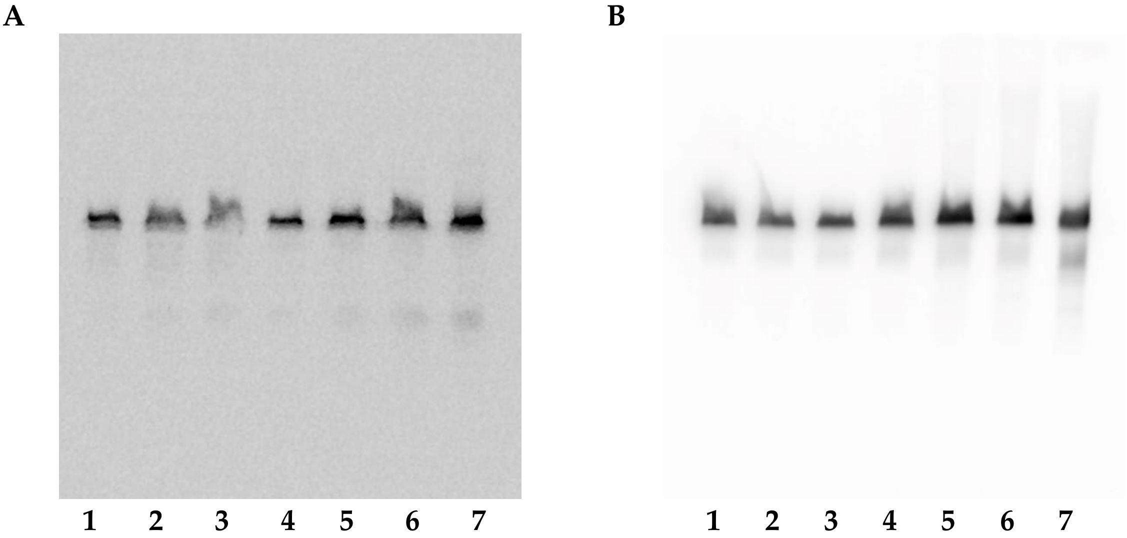

Figure S4. Original western blot membranes used for the evaluation of p53 expression in MCF-7 (**A**) and MDA-MB-231 (**B**) breast cancer cells. 1) Control; 2) Cisplatin 0.5 μM; 3) Cisplatin 1 μM; 4) EDAG-1 0.5 μM; 5) EDAG-1 1 μM; 6) EDAG-8 0.5 μM; 7) EDAG-8 1 μM.

A

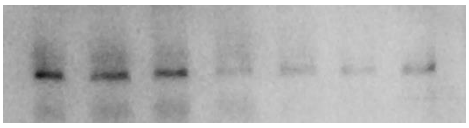

1 2 3 4 5 6 7

B

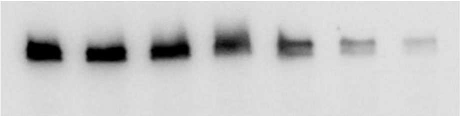

1 2 3 4 5 6 7

Figure S5. Original western blot membranes used for the evaluation of Akt expression in MCF-7 (**A**) and MDA-MB-231 (**B**) breast cancer cells. 1) Control; 2) Cisplatin 0.5 μM; 3) Cisplatin 1 μM; 4) EDAG-1 0.5 μM; 5) EDAG-1 1 μM; 6) EDAG-8 0.5 μM; 7) EDAG-8 1 μM.

A

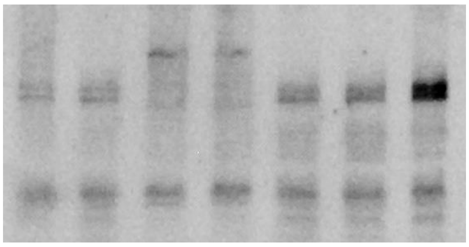

1 2 3 4 5 6 7

B

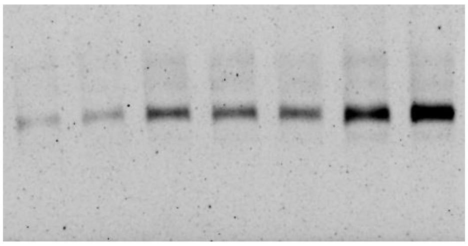

1 2 3 4 5 6 7

Figure S6. Original western blot membranes used for the evaluation of AMPK expression in MCF-7 (**A**) and MDA-MB-231 (**B**) breast cancer cells. 1) Control; 2) Cisplatin 0.5 μM; 3) Cisplatin 1 μM; 4) EDAG-1 0.5 μM; 5) EDAG-1 1 μM; 6) EDAG-8 0.5 μM; 7) EDAG-8 1 μM.

A

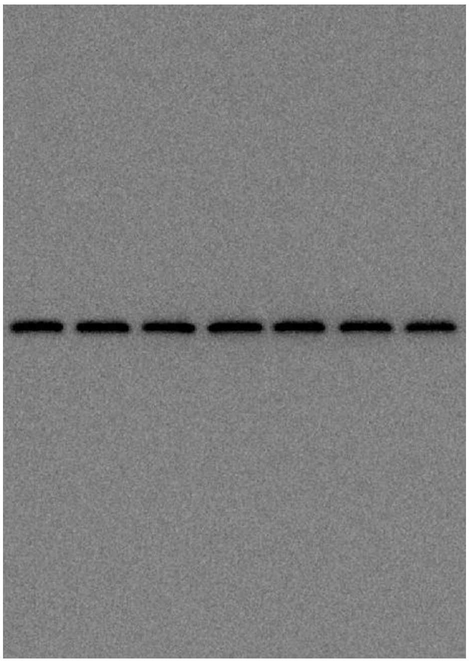

1 2 3 4 5 6 7

B

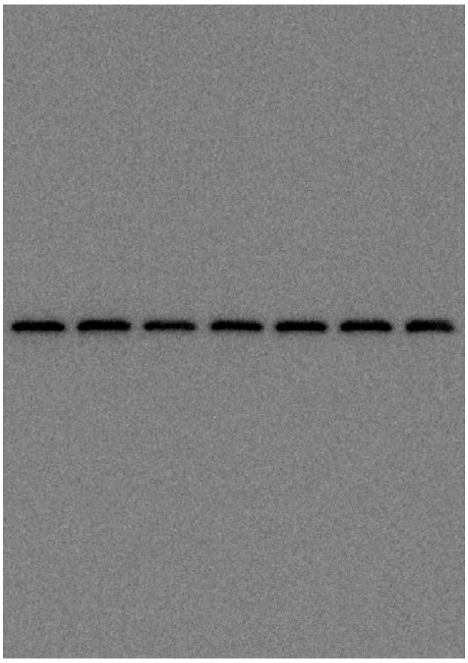

1 2 3 4 5 6 7

Figure S7. Original western blot membranes used for the evaluation of β-actin expression in MCF-7 (**A**) and MDA-MB-231 (**B**) breast cancer cells. 1) Control; 2) Cisplatin 0.5 μM; 3) Cisplatin 1 μM; 4) EDAG-1 0.5 μM; 5) EDAG-1 1 μM; 6) EDAG-8 0.5 μM; 7) EDAG-8 1 μM.
